# BREAST CANCER SCREENING AMONG OLDER NON-HISPANIC CARIBBEAN BLACK WOMEN IN THE UNITED STATES

**DOI:** 10.1093/geroni/igad104.2713

**Published:** 2023-12-21

**Authors:** Donnette Narine, Takashi Yamashita

**Affiliations:** University of Maryland Baltimore, Baltimore, Maryland, United States; University of Maryland, Baltimore County, Baltimore, Maryland, United States

## Abstract

Breast cancer is one of the most common forms of the second leading cause of death—cancer, which impacts racial and ethnic groups differently. The mortality rate of breast cancer among Black women is 40% higher than that of non-Hispanic White women. Early detection is critical for improving survival rates and reducing health disparities by race and ethnicity over the life course. However, the heterogeneity (i.e., within-group differences) among Black women in the United States is often overlooked, and relatively little is known about specific sub-populations of Black women and breast cancer screening. Of those sub-populations, Afro-Caribbean women in the United States tend to have different demographic and cultural backgrounds (e.g., a higher % of immigrants), for example, from African/African American women. Thus, this study examined factors associated with breast cancer screening among non-Hispanic Caribbean Black women. Using seven waves (2011-2017) of the National Health Interview Surveys data, we analyzed a sample of non-Hispanic Caribbean Black women living in the United States (age 40-75 years; n = 552). The results of the binary logistic regression indicated that the years in the United States were positively associated with breast cancer screening in the past 12 months among non-Hispanic Caribbean Black women (odds-ratio = 1.39, standard-error = 0.15, p < 0.05), after adjusting for the selected covariates. However, age was not associated with breast cancer screening behaviors. Our findings support breast cancer prevention efforts specifically targeting middle-aged and older non-Hispanic Caribbean Black women who have newly immigrated to the United States.

